# Delineating effect of corn microRNAs and matrix, ingested as whole food, on gut microbiota in a rodent model

**DOI:** 10.1002/fsn3.1672

**Published:** 2020-06-23

**Authors:** Haiqiu Huang, Quynhchi Pham, Cindy D. Davis, Liangli Yu, Thomas T.Y. Wang

**Affiliations:** ^1^ Diet, Genomics and Immunology Laboratory Beltsville Human Nutrition Research Center USDA‐ARS Beltsville Maryland USA; ^2^ Office of Dietary Supplements NIH Bethesda Maryland USA; ^3^ Department of Nutrition and Food Science University of Maryland College Park Maryland USA

**Keywords:** corn, dietary miRNAs, *Firmicutes*, gut microbiota, plant

## Abstract

Dietary microRNAs (miRNAs) are thought to regulate a wide range of biological processes, including the gut microbiota. However, it is difficult to separate specific effect(s) of miRNA from that of the food matrix. This study aims to elucidate the specific effect(s) of dietary corn miRNAs, ingested as a whole food, on the gut microbiota. We developed an autoclave procedure to remove 98% of miRNA from corn. A mouse feeding study was conducted comparing autoclaved corn to nonautoclaved corn and purified corn miRNA. Compared to nonspecific nucleotides and corn devoid of miRNAs, feeding purified corn miRNAs or corn to C57BL/6 mice via gavage or diet supplementation for two weeks lead to a decrease in total bacteria in the cecum. The effect appeared to be due to changes in *Firmicutes*. Additionally, corn matrix minus miRNA and processing also affected gut bacteria. In silico analysis identified corn miRNAs that aligned to *Firmicutes* genome sequences lending further support to the interaction between corn miRNAs and this bacterium. These data support interactions between plant food miRNA, as well as matrix, and the gut microbiota exist but complex. However, it provides additional support for mechanism by which bioactive dietary components interact with the gut microbiota.

## Graphical abstract

We developed a method to remove miRNA from food (corn) and use it to elucidate the effect of miRNA on gut microbiome in‐vivo. We found corn miRNA specifically decrease abundance of Firmicutes, while the matrix devoid of miRNA appeared to modulate Lactobacillus and Akkermansia muciniphila. Also, processing may affect bacteria such as Ruminococcus, Enterobacteriaceae. Hence, interactions between plant food miRNA, food matrix, and the gut microbiota exist but may be complex.

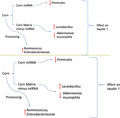

## 1 INTRODUCTION

A plant‐based diet is well documented at being protective against chronic diseases, such as cardiovascular diseases and cancer (Hill, Fleming, & Kris‐Etherton, 2009; Kris‐Etherton, Eckel, Howard, St Jeor, & Bazzarre, 2001; Meadows, 2012; Willett, 1995), and the beneficial health effects are often attributed to their low‐caloric and diverse chemical composition, such as the presence of fiber and phytochemicals (Babio, Balanza, Basulto, Bullo, & Salas‐Salvado, 2010; Lila & Raskin, 2005; Meadows, 2012). However, bioactive food components administered alone were often not able to achieve the same potency as complete plant materials (Witwer & Hirschi, 2014). This suggests that our current understanding of bioactive dietary components may not be complete, and additional bioactive components or interactions may exist.

Small RNAs are a class of small, functional, nonprotein‐coding RNAs that ubiquitously exist in microorganisms, plants, and animals, which are estimated to mediate 30% of the posttranscriptional silencing in mammals (Bartel, 2004; He & Hannon, 2004). Small RNAs are known to modulate a wide range of critical biological processes, including neuronal development, cell differentiation, apoptosis, proliferation, and immune response. Dysregulation of small RNAs is involved in various diseases, such as inflammation, Alzheimer's disease, and cancer (Baer, Claus, & Plass, 2013; Bartel, 2004; He & Hannon, 2004; Ranjha & Paul, 2013; Satoh, 2012). Recently, high levels of microRNAs (miRNAs) were detected in human breast milk and had the ability to survive at low pH environment and RNase present in the digestive tract, indicating that breast milk miRNAs may be designed to be accessible to infants through oral intake (Kosaka, Izumi, Sekine, & Ochiya, 2010; Munch et al., 2013). Other studies have shown that extracellular small RNAs are usually packaged, such as encapsulation in phospholipid bilayer vesicles or exosomes (Valadi et al., 2007), formation of small RNA–protein complexes (Gupta, Bang, & Thum, 2010), or high‐density lipoprotein (Vickers, Palmisano, Shoucri, Shamburek, & Remaley, 2011), which render these small RNAs extraordinarily stable and resistant to degradation in harsh environmental conditions, such as acidic pH and substantial heat exposure (Jung et al., 2010).

In 2012, the cross‐kingdom transfer of rice miRNAs (miR156a and miR168a) to human and mouse via dietary consumption was first reported (Zhang, Hou, et al., 2012). These exogenous miRNAs were reported to be at detectable levels in both blood and tissues, and miR168a was found to directly down‐regulate expression of the cholesterol regulation‐related gene LDLRAP1 in the liver (Zhang, Hou, et al., 2012). However, the notion of plant small RNAs surviving the digestive process and being present in the circulation at high enough concentrations to regulate tissue gene expression remains controversial and warrants further investigation (Beatty et al., 2014; Chin et al., 2016; Dickinson et al., 2013; Jiang, Sang, & Hong, 2012; Liang et al., 2012; Liang, Zen, Zhang, Zhang, & Chen, 2013; Lukasik & Zielenkiewicz, 2014; Snow, Hale, Isaacs, Baggish, & Chan, 2013; Vaucheret & Chupeau, 2012; Wang et al., 2012; Witwer, McAlexander, Queen, & Adams, 2013; Witwer, 2015; Yang, Farmer, Agyekum, Elbaz‐Younes, & Hirschi, 2015; Zhang, Hou, et al., 2012).

The relationship and putative effects of the gut microbiota on human health have attracted considerable attention (Cani, 2013), and a recent report on ginger‐derived miR7267‐3p regulating *Lactobacillus rhamnosus* monooxygenase ycnE expression further supported such potential interactions (Teng et al., 2018). The gut microbiota forms the first interface between the diet and the human body (Cho & Blaser, 2012). Therefore, the interaction between components in diet and the gut microbiota may play a critical role in how diet may contribute to or influence the pathology and development of chronic diseases, such as cardiovascular disease, obesity, and diabetes.

Based on the current understanding of the relationship between miRNAs and gut microbiota, dietary small RNAs may modulate the composition of human gut microbiota. However, studies using whole food to delineate the contribution of miRNAs, or other components in the food matrix, on gut microbiome remain scarce. In this study, we developed a method to remove miRNA in corn to serve as a matrix control to test the specific effects of corn miRNA. Corn was chosen in this study as the source of plant miRNAs as it is widely consumed by humans and is also a major component in the rodent diet. In vivo models were then employed to examine the interaction between corn miRNAs and the gut microbiota. Bioinformatic tools were also used to identify potential target sequences of corn miRNAs in the bacterial genome.

## 2 MATERIALS AND METHODS

### 2.1 Reagents and chemicals

Tris hydrochloride (pH 9.0), sodium dodecyl sulfate (SDS), lithium chloride (LiCl), ethylenediaminetetraacetic acid (EDTA), phenol (pH 8.0), chloroform–isoamyl alcohol (24:1; v/v), phenol–chloroform–isoamyl alcohol (25:24:1; v/v/v), sodium acetate (3 M, pH 5.2), sodium chloride (NaCl), polyethylene glycol 8,000, and pepsin were obtained from Sigma‐Aldrich (St. Louis, MO). RNA oligos with 3′ end 2′‐*O*‐methylation were synthesized by Integrated DNA Technologies, Inc. (Coralville, IA).

### 2.2 Plant small RNA isolation

Corn small RNAs were isolated according to a previously published protocol (Rosas‐Cardenas et al., 2011). Briefly, 0.1 g of pulverized plant sample was added to a 1.5 ml microcentrifuge tube with 500 μl of LiCl extraction buffer and 500 μl of phenol pH 8.0. The extraction mixture was vortexed for 1 min and incubated for 5 min at 60°C. Then, the mixture was centrifuged at 20,000 g at 4°C for 10 min. The upper phase was transferred to a new microcentrifuge tube, and 600 μl of chloroform–isoamyl alcohol (24:1; v/v) was added. The mixture was vortexed and centrifuged, and the upper phase was transferred to a new microcentrifuge tube and incubated at 65°C for 15 min. Then, 50 μl of 5 M NaCl and 63 μl of 40% polyethylene glycol 8,000 (w/v) were added followed by incubation on ice for at least 30 min. The low‐molecular‐weight RNA was separated from the pellet, which consisted of high‐molecular‐weight RNA and DNA by centrifugation. The supernatant was mixed with 500 μl of phenol–chloroform–isoamyl alcohol (25:24:1; v/v/v). The mixture was centrifuged, and the supernatant was transferred to a new microcentrifuge tube with 50 μl of 3 M sodium acetate pH 5.2 and 1,200 μl of absolute ethanol. The RNA sample was incubated at −20°C overnight. Small RNA was precipitated, washed twice, and dried. Isolated RNA was resuspended in RNase‐free water and kept at −80°C. RNA concentration and purity were determined using a Nanodrop 8000 Spectrophotometer (Thermo Fisher Scientific, St. Louis, MO).

### 2.3 Plant miRNA detection

The plant miRNAs were treated with periodate oxidation to confirm the 2‐*O*‐methylation at the 3′ end and detected using qRT‐PCR according to a previously published protocol (Huang, Roh, Davis, & Wang, 2017). TaqMan MicroRNA Assays (Table 1) were purchased from Thermo Fisher Scientific (St. Louis, MO) and used for microRNA reverse transcription and detection. TaqMan MicroRNA Reverse Transcription Kit (Thermo Fisher Scientific, St. Louis, MO), the small RNA‐specific RT primer from the TaqMan MicroRNA Assays, and 5 µl of 2 ng/µl RNA were used to reverse transcribe complementary DNA. Quantitative RT‐PCR (qRT‐PCR) was performed on ViiA7 Real‐time PCR System (Applied Biosystems, Foster City, CA) using 1 µl reverse transcription product, TaqMan Universal PCR Master Mix (Cat No.: 4304437), and small RNA‐specific TaqMan primer from the TaqMan MicroRNA Assays. The following amplification parameters were used for PCR: 50°C for 2 min, 95°C for 10 min, and 40 cycles of amplification at 95°C for 15 s and 60°C for 1 min. A 0.005 amol to 500 fmol range of synthetic miRNAs was used to construct a standard curve for qRT‐PCR.

**Table 1:** TaqMan MicroRNA assays

miRNA	Assay ID	Target miRNA sequence (5′ to 3′)
ath‐miR156a	000,333	UGACAGAAGAGAGUGAGCAC
ath‐miR164a	000,344	UGGAGAAGCAGGGCACGUGCA
zma‐miR166a	243607_mat	GGAAUGUUGUCUGGCUCGGGG
ath‐miR167a	000,348	UGAAGCUGCCAGCAUGAUCUA
zma‐miR168a	007594_mat	UCGCUUGGUGCAGAUCGGGAC
zma‐miR169p	243581_mat	GGCAAGUCAUCUGGGGCUACG
zma‐miR171j	241641‐mat	UAUUGACGCGGUUCAAUUCGA

### 2.4 Animals and diets

#### 2.4.1 Animals

Male C57BL/6 mice (5‐week old, Charles River, Wilmington, MA) were acclimated for a week with free access to water and AIN‐93M diet (D10012M, Research Diet, Inc., New Brunswick, NJ). Animals were single‐housed in ventilated racks for the duration of the experiment. The animal facility is specific pathogen‐free with a 12/12‐hr light and dark cycle and controlled at 23 ± 3°C and humidity between 30% and 70%.

#### 2.4.2 MiRNA containing and miRNA less corn

Corn depleted of miRNA was prepared according to our pilot study's results. Corn kernel was treated at 121°C for 30 min to degrade the miRNAs in the plant material. Corn kernel powder (nonautoclaved or autoclaved) was incorporated into animal's diets as ground powders (Table S1).

#### 2.4.3 Feeding study

After acclimation, mice (*n* = 10/group) were assigned to the following three groups for a feeding period of two weeks: (a) control (AIN‐93M diet); (b) AIN‐93M + random nucleotides; and 3) AIN‐93M + purified small RNA isolated from corn kernels. Random nucleotides and corn small RNA (25 μg in 100 µl) were administered by gavage using water as a vehicle at the beginning of the dark hours, and the control group was given 100 µl water. For experiment 2, mice (*n* = 10/group) were assigned to the following three diets for a feeding period of two weeks: (a) control (AIN‐93M diet); (b) AIN‐93M + 3% autoclaved corn kernel powder; and (c) AIN‐93M + 3% fresh corn kernel powder. The amounts of corn small RNA or corn kernel were based on 1 serving/day of 166 g corn kernel for human consumption (National Nutrient Database for Standard Reference, Release 28, Agricultural Research Service—USDA) and a 5‐g diet per day for mouse consumption. The powder was formulated into the diet by Research Diets, Inc. (New Brunswick, NJ). Animal diets were stored at 4°C throughout the feeding period. Fecal samples were collected daily. At the end of the feeding period, blood was collected by cardiac puncture with syringes previously rinsed in potassium EDTA solution (15% w/v) and kept on ice. Contents of the cecum, colon, and liver samples were collected and immediately frozen in liquid nitrogen. All samples were kept at −80°C before analysis. This study was carried out in strict accordance with the recommendations in the Guide for the Care and Use of Laboratory Animals of the National Institutes of Health. The protocol was approved by the U.S. Department of Agriculture (USDA) Agricultural Research Service (ARS) Beltsville Area Institutional Animal Care and Use Committee (IACUC) (Protocol # 16‐017).

### 2.5 Bacterial DNA extraction and PCR analysis for specific bacteria

Fecal pellets from the animal studies were homogenized with Precellys at 7,500 rpm for 1 min (Bertin Technologies, France). Bacterial DNA was extracted using a QIAmp DNA Stool Mini Kit from Qiagen following the manufacturer's protocol with modifications as previously described (Mirsepasi et al., 2014). DNA was eluted from the column with 100 μL nuclease‐free water. The concentration of DNA elution was determined by its absorbance at 260 nm, followed by serial dilutions to the final concentration of 10 ng/ml. Real‐time PCR was performed with a reaction system of 10 μl SYBR® Green Real‐Time PCR Master Mix, 0.205 μl 500 nM custom‐made oligo primers, 4.58 μl water, and 5 μl DNA. Primers specific for *Bacteroidetes* and *Firmicutes* phyla, *Akkermansia muciniphila* and *Bifidobacteria*, *Lactobacillus*, *Enterobacteriaceae*, *Ruminococcus*, and *Prevotella* genera were used to determine the relative abundance of respective microorganisms (Parnell & Reimer, 2012; Wang et al., 2014; Table S2).

#### 2.5.1 Bioinformatic analysis of miRNA–bacteria interaction

To assess the potential interaction between plant miRNAs commonly found in human and mouse diet and bacteria, the sequences of the abundant plant miRNAs were obtained from miRbase (Kozomara & Griffiths‐Jones, 2011). The abundant miRNAs reported for soy and tomato (Zhang et al., 2010) were also used in this analysis as a comparison with corn. The sequences were queried using NCBI BLASTN 2.6.0 + against bacteria genome for alignment to candidate bacterial genes. The respective target genes were searched in GenBank and KEGG database for the potential functionality of the encoded proteins.

### 2.6 Statistical analysis

Linear regression and statistical analysis were performed using GraphPad Prism 6 (2015, GraphPad Software, La Jolla, CA). Significance differences between means from treated groups compared to controls were determined using one‐way ANOVA and to allow for all possible pairwise comparisons whether sample sizes are equal, unequal, or with or without confidence intervals. Student's *t* test or Tukey's honestly significant difference (HSD) test was used in this study. Statistical significance was defined at *p* ≤ .05.

## 3 RESULTS

### 3.1 Quantitative analysis of selected miRNAs in corn and rodent chow by qRT‐PCR

**Figure 1:**
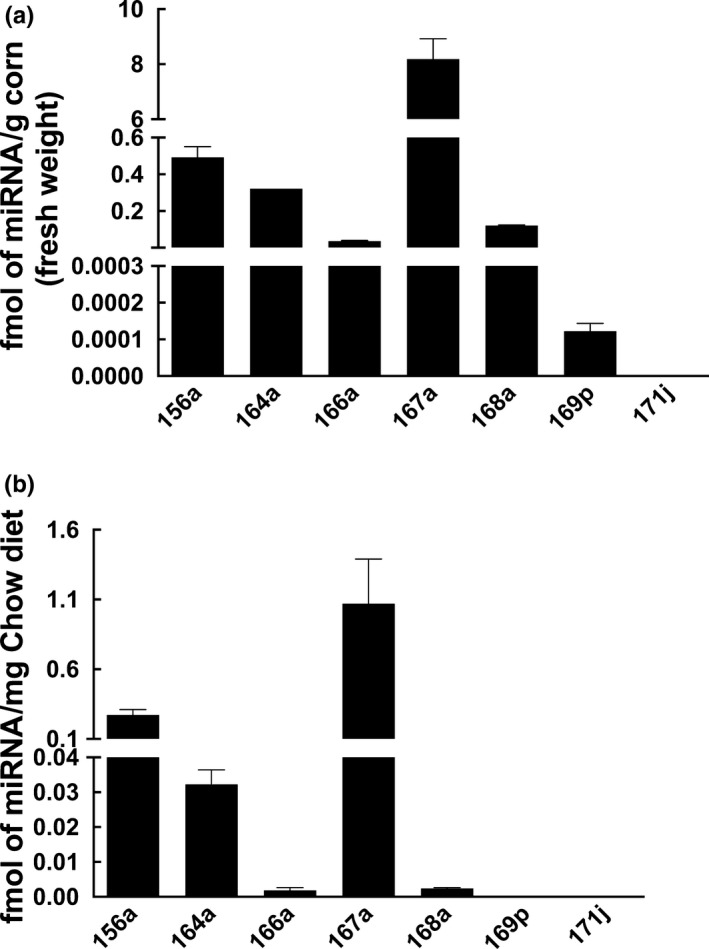
Detection of miRNAs in corn and rodent chow. Low‐molecular‐weight RNA was isolated from (a) fresh corn or (b) rodent chow as described in the Materials and Methods. Quantification of miR156a, miR164a, miR166a, miR167a, miR168a, miR169p, and miR171j was performed with TaqMan MicroRNA Assays using qRT‐PCR (data presented as Mean ± *SD*)

The levels of previously reported major plant miRNAs (miR156a, miR164a, miR166a, miR167a, miR168a, miR169p, and miR171j; Zhang et al., 2009) in fresh corn were analyzed and quantified. The concentration of these miRNAs ranged between 0.0001 and 8.19 fmol per gram of fresh corn, among which miR167a was the most abundant, and miR171j was undetectable in corn (Figure 1a). Rodent chow (8728C Teklad Certified Rodent Diet, Harlan Laboratories, Inc, Frederick, MD, USA), a corn–soy based diet, was also analyzed for its miRNA composition. Similarly, miR167a was determined to be the most abundant miRNA in the rodent chow at 1.07 fmol per gram, and miR169p and miR171j were not detected (Figure 1b).

### 3.2 Removal of miRNA by autoclaving

**Figure 2:**
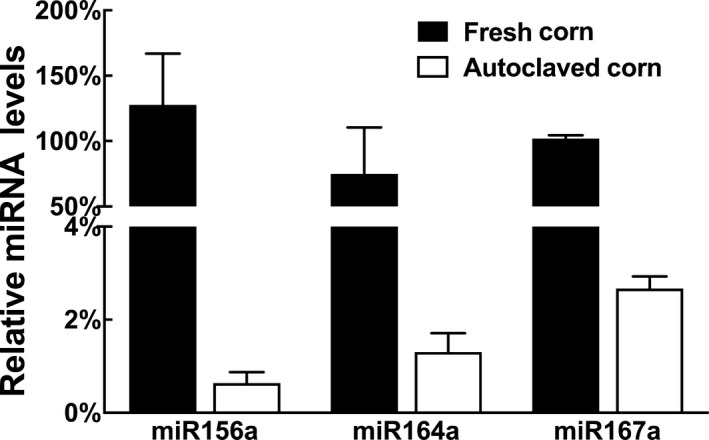
Degradation of corn miRNAs after autoclaving. Fresh corn kernels were autoclaved at 121°C for 30 min. Then, miRNAs were extracted from fresh corn kernels or autoclaved corn kernels. The relative concentrations of miR156a, miR164a, and miR167a were determined using qRT‐PCR (data presented as Mean ± *SD*)

**Figure 3:**
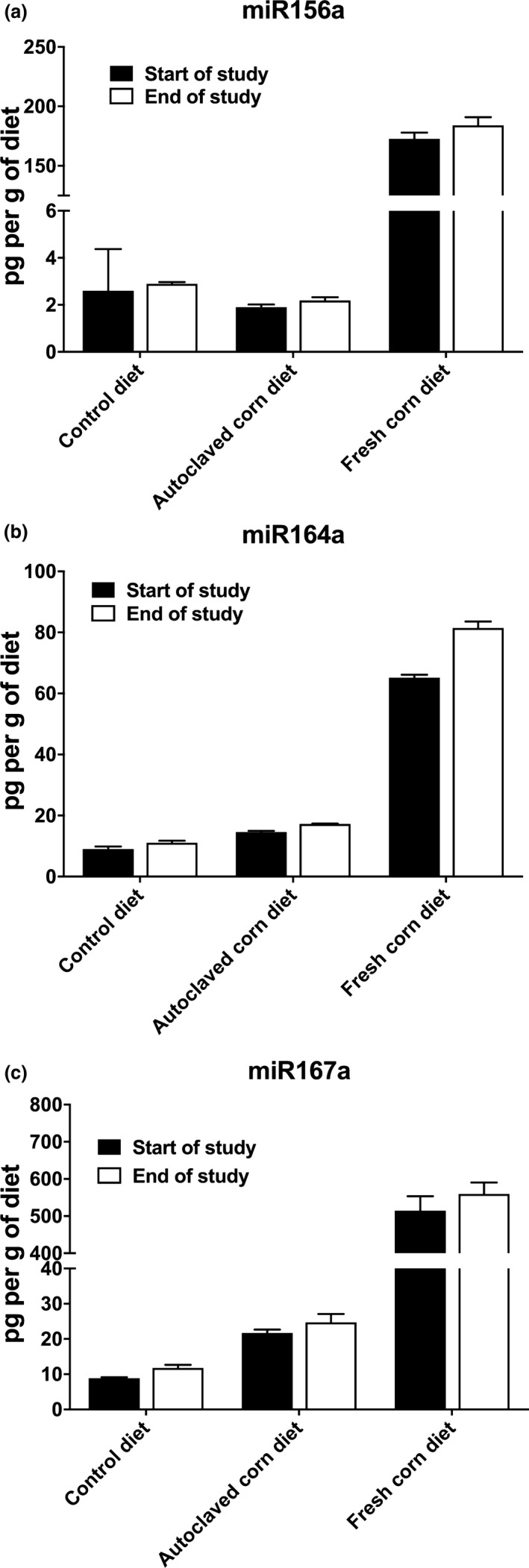
Stability of corn miRNAs in AIN‐93M diet at the start and end of the feeding period. MiRNAs were extracted from AIN‐93M diets at the start or end of the feeding period. The relative concentrations of (a) miR156a, (b) miR164a, and (c) miR167a were determined using qRT‐PCR (data presented as Mean ± *SD*)

Autoclaving the corn sample at 121°C for 30 min was shown to degrade more than 98% of miRNAs (Figure 2). The autoclaved corn, depleted of miRNAs, served as a control for macronutrients and food matrix provided by the 3% fresh corn powder supplementation. The corn miRNAs in the AIN‐93M diet were determined to be stable throughout the two‐week feeding period (Figure 3a, b, c).

### 3.3 In vivo effect of corn miRNAs, autoclaved corn, and fresh corn on mouse gut microbiota

C57BL/6 mice (5‐week old, 10 per group) were (a) gavaged with 25 µg of random dNTPs or corn miRNAs per day or (b) fed with AIN‐93M diet supplemented with 3% (w/w) fresh or autoclaved corn powder. No difference was observed in body weight and food intake in both animal experiments throughout the 2‐week feeding period (data not shown). At the end of the feeding period, cecum content was analyzed for bacteria composition using PCR.

**Figure 4:**
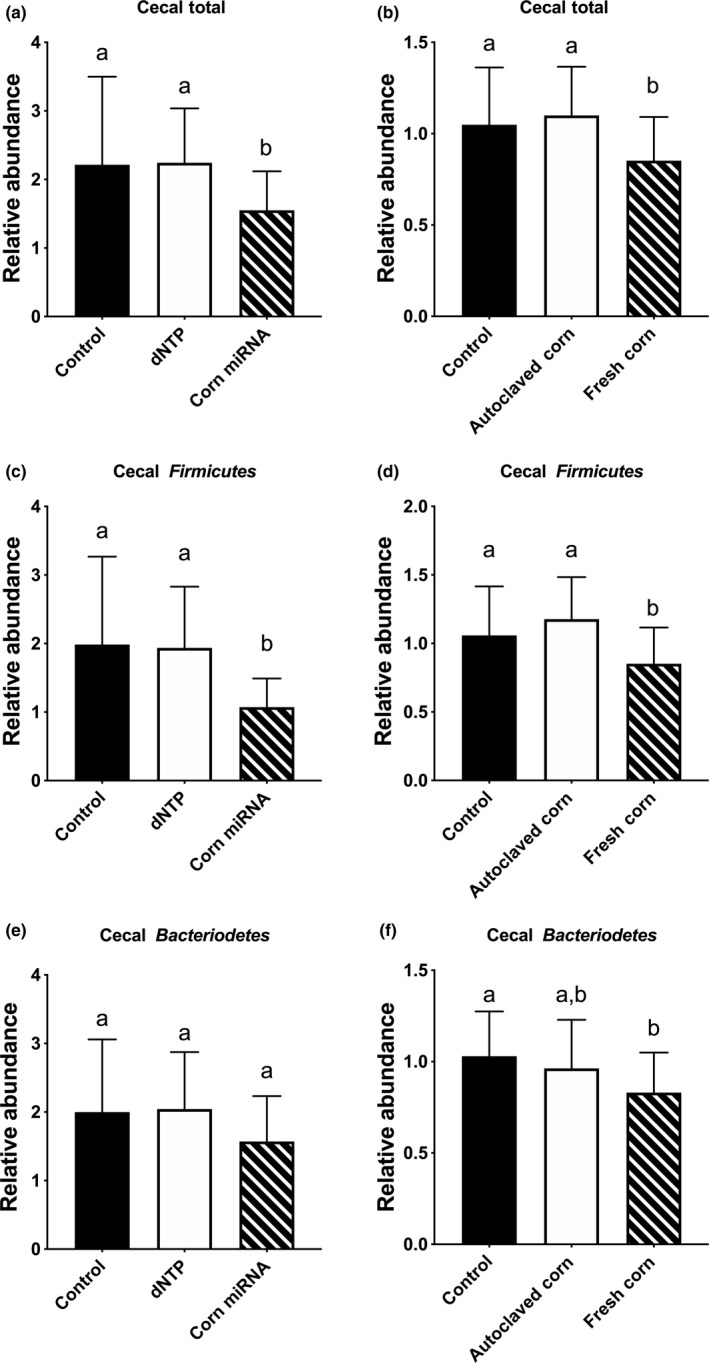
In vivo effects of corn miRNA, autoclaved corn and fresh corn on total bacteria, *Firmicutes*, and *Bacteroidetes*. Corn miRNA extracts (25 µg per day) (a, c, e) or AIN‐93M diet supplemented with 3% (w/w) corn powder (b, d, f) was given to C57BL/6 mice for two weeks. At the end of the feeding period, cecum content was collected and cecal bacteria DNA isolated and purified as described in the Materials and Methods. Quantification of bacteria was performed with specific primers using qRT‐PCR. Data were presented as relative abundance comparing to that of the control group. Columns marked with different letters are significantly different from each other (*p* ≤ .05, Mean ± *SD*). (a, b) Total bacteria, (c, d) *Firmicutes,* and (e, f) *Bacteroidetes*

**Figure 5:**
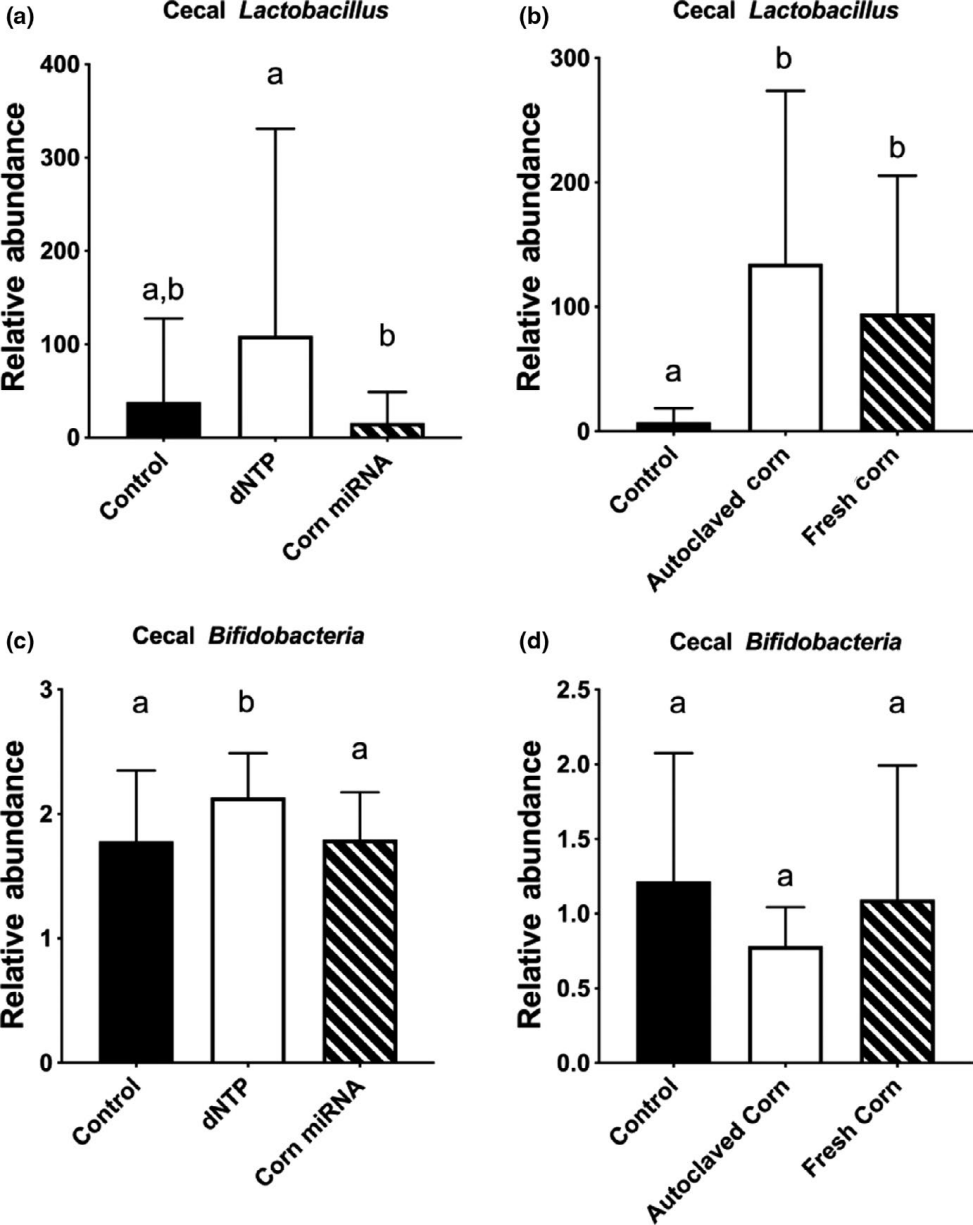
In vivo effects of corn miRNA, autoclaved corn and fresh corn on *Lactobacillus*, and *Bifidobacteria*. Corn miRNA extracts (25 µg per day) (a, c) or AIN‐93M diet supplemented with 3% (w/w) corn powder (b, d) was given to C57BL/6 mice for two weeks. At the end of the feeding period, cecum content was collected and DNA was isolated and purified as described in the Materials and Methods. Quantification of bacteria was performed with specific primers using qRT‐PCR. Data were presented as relative abundance comparing to that of the control group. Columns marked with different letters are significantly different from each other (*p* ≤ .05, Mean ± *SD*). (a, b) *Lactobacillus*, and (c, d) *Bifidobacteria*

**Figure 6:**
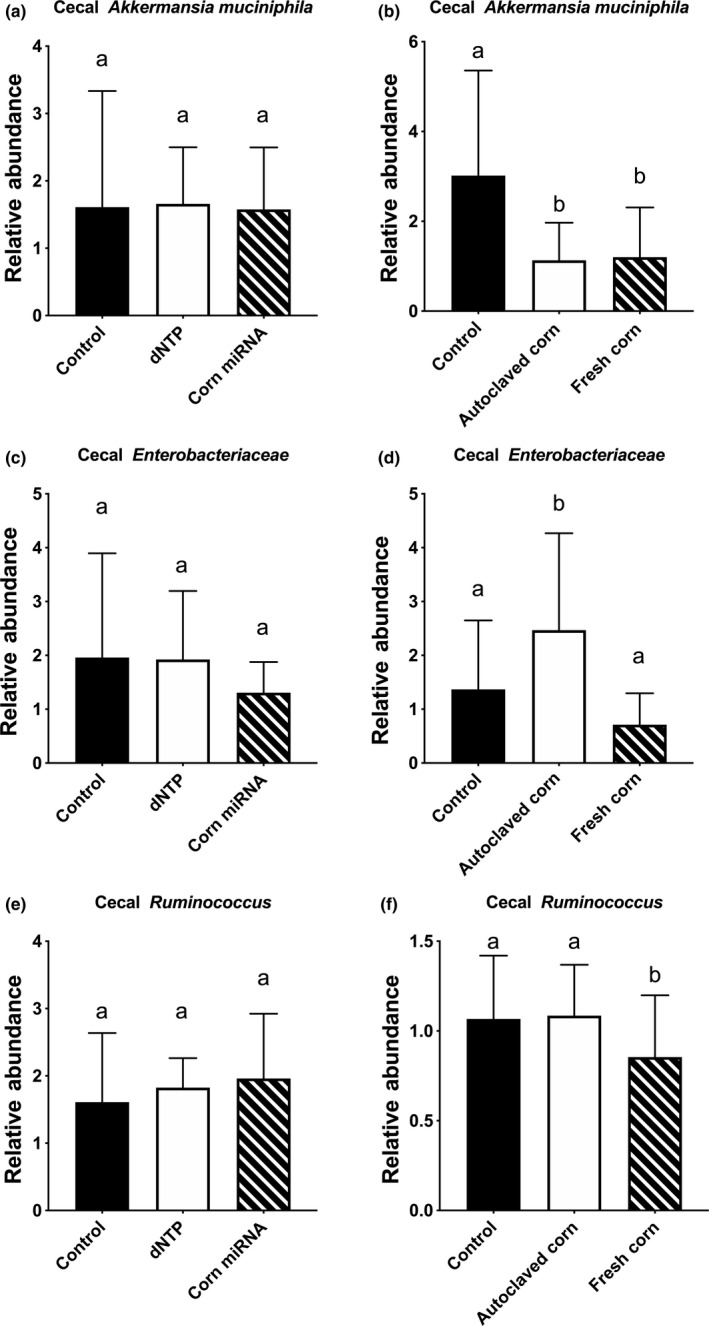
In vivo effects of corn miRNA, autoclaved corn and fresh corn on *Akkermansia muciniphila*, *Enterobacteriaceae*, and *Ruminococcus*. Corn miRNA extracts (25 µg per day) (a, c, e) or AIN‐93M diet supplemented with 3% (w/w) corn powder (b, d, f) was given to C57BL/6 mice for two weeks. At the end of the feeding period, cecum content was collected and DNA was isolated and purified as described in the Materials and Methods. Quantification of bacteria was performed with specific primers using qRT‐PCR. Data were presented as relative abundance comparing to the control group. Columns marked with different letters are significantly different from each other (*p* ≤ .05, Mean ± *SD*). (a, b) *Akkermansia muciniphila*, (c, d) *Enterobacteriaceae,* and (e, f) *Ruminococcus*

We first compared the total bacterial abundance. We found that total bacterial abundance in cecal samples of corn miRNAs administered via gavage or fresh corn‐supplemented diet to be significantly lower than dNTP control or autoclaved corn control (Figure 4a, b). We further compared the two major gut bacteria phylum *Firmicutes* and *Bacteroidetes*. We found that *Firmicutes* in the cecal samples was lower than the dNTP or autoclaved corn control groups, respectively (Figure 4c, d). By contrast, no differences were detected in *Bacteroidetes* when comparing the corn miRNA, fresh corn groups to the dNTP or autoclaved corn controls (Figure 4e, f). However, a slight but significant lower *Bacteroidetes* was observed when comparing fresh corn to the control nonsupplemented diet (Figure 4f). We further investigated the effects on selected biologically important bacteria reported in the literature. We found no difference between autoclaved corn and fresh corn, but both groups had a higher abundance of *Lactobacillus* than unsupplemented animals (Figure 5b). Also, the corn miRNA‐gavaged group has significantly lower *Lactobacillus* than the dNTP control (Figure 5a). In addition, dNTP administration appeared to increase *Bifidobacterium* as compared to control and corn miRNA‐gavaged cecal samples (Figure 5c) but not corn (Figure 5d). We found no differences in the genus of *Bacteroidetes*, known to be associated with a plant‐based diet (Lossaso et al., 2018), between the different diet groups (data not shown). The bacteria *Akkermansia muciniphila* is known to be associated with chronic diseases such as obesity and diabetes (Depommier et al., 2019; Everard et al., 2013). We found no difference between corn miRNA and dNTP control (Figure 6a). In contrast, both autoclaved corn and fresh corn have lower *A. muciniphila* than unsupplemented control (Figure 6b). *Enterobacteriaceae*, which includes many of bowel infection related bacteria, like *E. coli* was also examined. We found that autoclaved corn, in particular, has a high abundance of *Enterobacteriaceae* than fresh corn and control group (Figure 6c, d). *Ruminococcus*, another genus of bacteria associated with inflammatory bowel, was also examined. Fresh corn group, in particular, has a lower abundance of this genus when compared to autoclaved corn and unsupplemented control (Figure 6e, f).

### 3.4 Bioinformatic analysis of plant miRNA target sequences in the bacterial genome

To complement our in vivo work, an in silico analysis was also performed to identify potential bacterial sequences miRNA can interact with. We identified 141–262 target genes through our in silico analysis (Table 2). One limitation is that the NCBI database includes the genome of all microbes, and therefore, the aligned sequences and corresponding species are not limited to the species found in the gut microbiota. Bacteria that are potentially targeted by plant miRNAs varied widely, and *Bacteroidetes*, *Firmicutes*, *Actinobacteria*, and *Proteobacteria* phyla accounted for most bacteria containing the target genes (Table 3). Further examination of the alignment data using criteria of high identity and no gaps, bacteria in *Firmicutes* and *Bacteriodetes* (Table 4) phyla were identified, which are the dominant phyla in gut microbiota. The predicted target proteins included PAS domain‐containing sensor histidine kinase, nucleotide pyrophosphatase, phosphocarrier protein HPr, 4‐hydroxybutyrate CoA‐transferase, cell division protein FtsW, ABC transporter ATP‐binding protein, sensor histidine kinase, and dihydrodipicolinate synthase, which are proteins or enzymes in the key metabolic or cell growth pathways.

**Table 2:** In silico analysis of plant miRNA target sequences in the bacteria genome

miRNA	Sequence	Query ID	Microbe Genome Blast Hits
zma‐miR156a	UGACAGAAGAGAGUGAGCAC	lcl|Query_126643	166
zma‐miR164a	UGGAGAAGCAGGGCACGUGCA	lcl|Query_152519	239
zma‐miR167a	UGAAGCUGCCAGCAUGAUCUA	lcl|Query_14779	224
gma‐miR167b	UGAAGCUGCCAGCAUGAUCUA	lcl|Query_10873	187
gma‐miR168a	UCGCUUGGUGCAGGUCGGGAA	lcl|Query_42237	262
sly‐miR164b	CACGUGUUCUCCUUCUCCAAC	lcl|Query_58715	235
sly‐miR168a	CCUGCCUUGCAUCAACUGAAU	lcl|Query_81397	141

Note

zma: corn miRNA; gma: soy miRNA; sly: tomato miRNA.

Sequence alignment of plant miRNAs with microbe genome was performed using NCBI BLASTN 2.6.0 + according to previously published manual. (Altschul et al., 1997).

**Table 3:** Major bacteria phyla containing target sequences

Phylum		# and percentage of hits					
	zma‐miR156a	zma‐miR164a	zma‐miR167a	gma‐miR167b	gma‐miR168a	sly‐miR164b	sly‐miR168a
*Actinobacteria*	6 hits, 7%^a^	38 hits, 44%	3 hits, 4%	3 hits, 4%	20 hits, 23%	34 hits, 48%	0 hits, 0%
*Bacteroidetes*	12 hits, 14%	6 hits, 7%	7 hits, 9%	5 hits, 6%	0 hits, 0%	3 hits, 4%	6 hits, 7%
*Firmicutes*	23 hits, 27%	6 hits, 7%	17 hits, 23%	20 hits, 27%	0 hits, 0%	17 hits, 24%	20 hits, 21%
*Proteobacteria*	32 hits, 38%	36 hits, 41%	44 hits, 60%	44 hits, 60%	51 hits, 58%	11 hits, 16%	53 hits, 58%
Others	11 hits, 14%	1 hit, 1%	3 hits, 4%	2 hits, 3%	17 hits, 19%	5 hits, 8%	13 hits, 14%

^a^ Percentage calculated as numbers of species containing sequences targeted by the plant miRNA in each phylum divided by a total number of bacteria species containing target sequences identified in NCBI Microbial genomes.

**Table 4:** Potential bacteria genes targeted by plant miRNAs

Phylum	Organism	Target function	Identities	Gaps	Sequence ID and range
zma‐miR156a					
*Firmicutes*	*Bacillus lehensis* G1	PAS domain‐containing sensor histidine kinase	14/14	0/14	NZ_CP003923.1 Range: 1,117,225–1,117,238
*Firmicutes*	*Brevibacillus laterosporus* LMG 15,441	Nucleotide pyrophosphatase	15/15	0/15	NZ_CP007806.1 Range: 2,458,733–2,458,747
*Firmicutes*	*Clostridium indolis* DSM 755	Phosphocarrier protein HPr	15/15	0/15	NZ_AZUI01000001.1 Range: 3,140,572–3,140,586
*Bacteroidetes*	*Flectobacillus major* DSM 103	4‐hydroxybutyrate CoA‐transferase	16/16	0/16	NZ_KE386491.1 ange: 89,592–89,607
*Bacteroidetes*	*Dokdonia donghaensis* DSW‐1	Cell division protein FtsW	13/13	0/13	NZ_JSAQ01000001.1 Range: 2,731,359–2,731,371
zma‐miR167a					
*Firmicutes*	*Bacillus simplex*	ABC transporter ATP‐binding protein	13/13	0/13	NZ_CP011008.1 Range: 1,133,803–1,133,815
*Firmicutes*	*Desulfosporosinus youngiae* DSM 17,734	Sensor histidine kinase	16/16	0/16	NZ_CM001441.1 Range: 1,900,368–1,900,383
*Bacteroidetes*	*Flectobacillus major* DSM 103	Dihydrodipicolinate synthetase	13/13	0/13	NZ_KE386491.1 Range: 1,221,927–1,221,939

## 4 DISCUSSION

In the present study, we confirmed our hypothesis that miRNAs from corn can modulate the gut microbiome. Moreover, we also identified corn matrix, as well as processing conditions, may all play roles in modulating the gut microbiome and, in some cases, overshadow the effects of miRNAs.

Interaction between food and the gut microbiome is complex. To ascribe a specific effect of a food component on the gut microbiome, one also needs to consider the relationship between a specific food component and the food matrix (i.e., other components). In this study, we used corn as the model and developed an autoclaving process to eliminate 98% of miRNAs. This allows us to have a matrix control to compare with and to identify the specific effect of corn miRNAs. To our knowledge, this type of work has not been reported. Recent literature, including one published by Teng et al. (2018) using ginger‐derived miR7267‐3p, supports the hypothesis that plant miRNA can modulate the gut microbiota and influence host physiology (Munch et al., 2013; Teng et al., 2018; Wang et al., 2012). However, studies such as the one by Teng et al. (2018) often utilize miRNA extracts or miRNAs packaged in exosome as the source of miRNA to test the effects of miRNA on the gut microbiota (Herwijnen et al., 2018; Teng et al., 2018; Zempleni, 2017). We have taken a different approach by eliminating miRNA while preserving the matrix of corn. Although we cannot completely eliminate the possibility that exosome may be affected, by using different controls, we concluded that corn miRNA appeared to modulate *Firmicutes* abundance in the gut. This notion is supported by observing that both corn miRNA and fresh corn have lower *Firmicutes* than dNTP and autoclaved corn controls. The lower *Firmicutes* abundance may contribute to the overall lower abundance of total bacteria observed in corn miRNA and fresh corn group. *Firmicutes* and *Bacteroidetes* are the two dominant phyla in gut microbiota (Turnbaugh et al., 2006), and an increase in *Firmicutes*, a decrease in *Bacteroidetes,* or alteration of their ratio have been reported to be associated with risk of obesity and other metabolic syndromes (Carmody et al., 2015; Turnbaugh, Backhed, Fulton, & Gordon, 2008). *Firmicutes* have also been shown to affect energy resorption, which in turn contribute to the development of diabetes and obesity, inflammation and other immune response, and risks of chronic diseases, such as cardiovascular diseases and cancers (Ley, Turnbaugh, Klein, & Gordon, 2006; Salonen & de Vos, 2014; Sekirov, Russell, Antunes, & Finlay, 2010; Turnbaugh et al., 2006; Young, 2012). Our result would be consistent with the health promotional effects of corn miRNA through regulation of *Firmicutes*.

When the analysis focused on the genus or species level, the effect of miRNA on bacteria appeared to be complicated by matrix. For example, *Lactobacillus* abundance was affected by corn miRNA as compared to dNTP, but both autoclaved corn and fresh corn actually increase the abundance of this genus of bacteria. Furthermore, *A. muciniphila* abundance was significantly lower in both autoclaved corn and fresh corn, but not corn miRNA, suggesting matrix may be a more important determinant for this bacterium. Hence, the wide range of other components exist in food may overshadow the effects of miRNAs on a specific bacterium. In addition to matrix, food processing may also influence the gut microbiome. This is exemplified by observing autoclaved corn, having a higher abundance of *Enterobacteriaceae* and *Ruminococcus*. It is possible that under other processing conditions, such as cooking that alters the composition of food, may alter microbiome modulatory property of food. The biological significance of the changes in the bacteria remains unclear and requires further elucidation and validation.

In silico analysis identified sequences in the bacterial genome that align with plant miRNAs using high identity and no gaps. Sequence complementarity serves as the molecular basis of miRNA actions (Chen, 2005; Ivashuta et al., 2009). As indicated in Table 4, plant miRNAs are predicted to target/interact with several functional proteins in the bacteria. The alignments’ results lend further support for regulation of gut bacteria by food‐derived miRNA and can also serve as the theoretical basis to explain how plant miRNAs may regulate gut microbiota; however, further elucidation is required.

Analysis of rodent chow, which is often derived from corn and soy, indicated that the small RNAs exist at detectable levels. Given that corn miRNAs and possibly other miRNAs may influence the gut microbiome, results from rodent experiments may be influenced by the types of diets the animals are fed. Using a synthetic purified diet, which does not contain miRNA, we expect that at least the abundance of *Firmicutes* would be different from animals fed a chow diet. Interpretation of microbiome data from dietary studies would need to be cautious regarding the correlation of diet, gut microbiome changes, and biological efficacies endpoints.

Observing the changes in bacteria may have health implication. As mentioned above, the abundance of *Firmicutes* is related to risk of obesity and metabolic diseases (Ley et al., 2006; Salonen & de Vos, 2014; Sekirov et al., 2010; Turnbaugh et al., 2006; Young, 2012). Hence, corn and/or corn miRNA may consider to be health promoting as they exhibit lower *Firmicutes* as compared to controls. However, lower abundance of *A. muciniphila* exerted by corn/corn matrix may be considered not healthy as higher abundance of these bacteria is considered protective against cardiovascular disease and diabetes (Depommier et al., 2019; Everard et al., 2013). Validation of these variable endpoints in the gut microbiota is necessary to further delineate health implication of corn and may be complex.

In conclusion, we demonstrated through using a matrix control that miRNA in food (i.e., corn) can modulate the gut microbiome. The bacteria phylum *Firmicutes* was identified as the target for corn miRNA in vivo. Given the reported role of *Firmicutes* in human health, a role for food‐derived miRNA in health promotion may be possible. Moreover, the food matrix, as well as processing, may also contribute to a complex interaction that can lead to changes in the gut microbiome.

## CONFLICT OF INTEREST

The authors declare no conflict of interest.

## STUDIES INVOLVING ANIMAL

The study's protocols and procedures were ethically reviewed and approved by the U.S. Department of Agriculture (USDA) Agricultural Research Service (ARS) Beltsville Area Institutional Animal Care and Use Committee (IACUC) (Protocol # 16‐017) in compliance with US National Research Council's Guide for the Care and Use of Laboratory Animals, the US Public Health Service's Policy on Humane Care and Use of Laboratory Animals, and Guide for the Care and Use of Laboratory Animals.

## Supporting information

Supplementary Material
