# Supplementary material for: Delineating effect of corn microRNAs and matrix, ingested as whole food, on gut microbiota in a rodent model
Source: Food Sci Nutr. 2020 Jun 23;8(8):4066–77. doi: 10.1002/fsn3.1672 (PMC7455949; doi:10.1002/fsn3.1672)
Supplement: Supplementary file 1 — Supplementary Material [file FSN3-8-4066-s001.docx]

SUPPORTING INFORMATION

Table 1 Diet composition for animal experiment 2

|  | **Control** | **Treatment** |
| --- | --- | --- |
| Ingredient | gm | gm |
| Casein | 140 | 135.9 |
| L-Cystine | 1.8 | 1.8 |
| Corn Starch | 495.692 | 473.8 |
| Maltodextrin 10 | 125 | 125 |
| Sucrose | 100 | 97 |
| Cellulose, BW200 | 50 | 47.5 |
| Soybean Oil | 40 | 39.1 |
| t-Butylhydroquinone | 0.008 | 0.008 |
| Mineral Mix S10022M | 35 | 35 |
| Vitamin Mix V10037 | 10 | 10 |
| Choline Bitartrate | 2.5 | 2.5 |
| Lyophilized fresh or autoclaved corn | 0 | 30 |
| **Total** | **1000** | **997.608** |
|  |  |  |
| **gm** |  |  |
| Protein | 125.0 | 125.0 |
| Fat | 40.0 | 40.0 |
| Total Carbohydrate | 730.7 | 730.7 |
| Sugar | 100.0 | 100.0 |
| Fiber | 50.0 | 50.0 |
| **kcal** |  |  |
| Protein | 500 | 500 |
| Fat | 360 | 360 |
| Total Carbohydrate | 2923 | 2923 |
| Sugar | 400 | 400 |
| Total Kcal | **3783** | **3783** |
| **kcal%** |  |  |
| Protein | 13 | 13 |
| Fat | 10 | 10 |
| Total Carbohydrate | 77 | 77 |
| Sugar | 11 | 11 |
| Total | 100 | 100 |
| **kcal/gm** | **3.78** | **3.79** |

Table 2 Sequence of Real-time PCR Primers

| **Bacteria** | **Direction** | **Sequence (5’-3’)** |
| --- | --- | --- |
| Total bacteria | Forward | ACTCCTACGGGAGGCAG |
|  | Reverse | GTATTACCGCGGCTGCTG |
| Bifidobacteria | Forward | TCGCGTCYGGTGTGAAAG |
|  | Reverse | CCACATCCAGCRTCCAC |
| Lactobacillus | Forward | GAGGCAGCAGTAGGGAATCTTC |
|  | Reverse | GGCCAGTTACTACCTCTATCCTTCTTC |
| Akkermansia | Forward | CAGCACGTGAAGGTGGGGAC |
|  | Reverse | CCTTGCGGTTGGCTTCAGAT |
| Prevotella | Forward | TCCTACGGGAGGCAGCAGT |
|  | Reverse | CAATCGGAGTTCTTCGTG |
| Enterobacteriaceae | Forward | CATTGACGTTACCCGCAGAAGAAGC |
|  | Reverse | CTCTACGAGACTCAAGCTTGC |
| Ruminococcus | Forward | GGCGGCCTACTGGGCTTT |
|  | Reverse | CCAGGTGGATAACTTATTGTGTTAA |
| Bacteroidetes | Forward | GGARCATGTGGTTTAATTCGATGAT |
|  | Reverse | AGCTGACGACAACCATGCAG |
| Firmicutes | Forward | GGAGYATGTGGTTTAATTCGAAGCA |
|  | Reverse | AGCTGACGACAACCATGCAC |
